# Generation, functional annotation and comparative analysis of black spruce (*Picea mariana*) ESTs: an important conifer genomic resource

**DOI:** 10.1186/1471-2164-14-702

**Published:** 2013-10-11

**Authors:** Ishminder K Mann, Jill L Wegrzyn, Om P Rajora

**Affiliations:** 1 Forest Genetics and Biotechnology Group, Department of Biology, Life Sciences Centre, Dalhousie University, 1355 Oxford Street, Halifax, NS B3H 4J1, Canada; 2 Faculty of Forestry and Environmental Management, University of New Brunswick, 28 Dineen Drive, P.O. Box 44000, Fredericton, NB E3B 5A3, Canada; 3 Department of Plant Sciences, University of California at Davis, One Shields Avenue MC4, Davis, California 95616, USA

**Keywords:** *Picea mariana*, Expressed sequence tag, Gene discovery, Gene expression, Gene ontology, Microsatellites

## Abstract

**Background:**

EST (expressed sequence tag) sequences and their annotation provide a highly valuable resource for gene discovery, genome sequence annotation, and other genomics studies that can be applied in genetics, breeding and conservation programs for non-model organisms. Conifers are long-lived plants that are ecologically and economically important globally, and have a large genome size. Black spruce (*Picea mariana*), is a transcontinental species of the North American boreal and temperate forests. However, there are limited transcriptomic and genomic resources for this species. The primary objective of our study was to develop a black spruce transcriptomic resource to facilitate on-going functional genomics projects related to growth and adaptation to climate change.

**Results:**

We conducted bidirectional sequencing of cDNA clones from a standard cDNA library constructed from black spruce needle tissues. We obtained 4,594 high quality (2,455 5*'* end and 2,139 3*'* end) sequence reads, with an average read-length of 532 bp. Clustering and assembly of ESTs resulted in 2,731 unique sequences, consisting of 2,234 singletons and 497 contigs. Approximately two-thirds (63%) of unique sequences were functionally annotated. Genes involved in 36 molecular functions and 90 biological processes were discovered, including 24 putative transcription factors and 232 genes involved in photosynthesis. Most abundantly expressed transcripts were associated with photosynthesis, growth factors, stress and disease response, and transcription factors. A total of 216 full-length genes were identified. About 18% (493) of the transcripts were novel, representing an important addition to the Genbank EST database (dbEST). Fifty-seven di-, tri-, tetra- and penta-nucleotide simple sequence repeats were identified.

**Conclusions:**

We have developed the first high quality EST resource for black spruce and identified 493 novel transcripts, which may be species-specific related to life history and ecological traits. We have also identified full-length genes and microsatellite-containing ESTs. Based on EST sequence similarities, black spruce showed close evolutionary relationships with congeneric *Picea glauca* and *Picea sitchensis* compared to other Pinaceae members and angiosperms. The EST sequences reported here provide an important resource for genome annotation, functional and comparative genomics, molecular breeding, conservation and management studies and applications in black spruce and related conifer species.

## Background

In non-model species with large genome size, EST (expressed sequence tag) sequencing and their annotation can provide the first step towards understanding the transcriptome and expression patterns of specific genes, which can complement the whole genome sequencing, and can assist with genome sequence annotation. Traditionally, EST sequencing was conducted with the Sanger sequencing system [1-5]. More recently, next-generation sequencing (NGS) platforms have been used to generate enormous amounts of genome and transcriptome sequences [6-10]. NGS methods facilitate whole transcriptome sequencing at a fraction of the time and cost previously required for the Sanger method [11,12]. However, commonly used NGS platforms produce shorter reads and/or reduce the quality per base call [13]. The improved length and accuracy of reads obtained from Sanger sequencing can complement NGS workflows. This technology can assist in validating the NGS platform sequences by serving as a reference by which short reads can be aligned and corrected [6]. Therefore, EST sequences derived from the Sanger method are still a valuable resource in the NGS era.

Conifers have a large genome size (~18-35 Gbp) and are ecologically and economically important, long-lived plants. They form a major part of the northern boreal and temperate forests, which constitute the major biome of the world. The genus *Pinus* (pine) and *Picea* (spruce) are two important genera among conifers. Black spruce (*Picea mariana* (Mill.) B.S.P.) is a widely-distributed transcontinental species of the North American boreal and temperate forests with high ecological and economic importance [14]. Black spruce is one of the most important softwood species for the production of pulp and paper in Canada [15]. It is an early successional species and has a corresponding suite of species-specific life history, growth, eco-physiological and adaptive traits [14]. The estimated haploid genome size of black spruce is about 17.5 Gbp, with 1C contents of 17.4 pg [16].

As of, January 1^st^, 2013 dbEST release (130101), there were approximately 74.19 million ESTs from 2,473 species available in the GenBank at the National Centre for Biotechnology Information (NCBI) [17]. In conifers, the major EST contributing species are loblolly pine - *Pinus taeda* (328,662), followed by white spruce - *Picea glauca* (313,110) and Sitka spruce - *Picea sitchensis* (186,637). Among spruce species, white spruce has the maximum number of ESTs, followed by *P. sitchensis* and *P. engelmannii* X *P. sitchensis.* Recently 27,720 unique cDNA clusters (unigene set) have also been reported for *P. glauca*[18]. Also, very recently, draft genomes of Norway spruce (*Picea abies*) and white spruce have been published [9,10]. However, the black spruce transcriptome is not yet fully characterized and only 4 ESTs and 699 cDNA sequences are reported within the NCBI’s dbEST (excluding ESTs reported from the current study). Due to a number of life history, morphological, adaptive, eco-physiological, and insect resistance traits and phylogenetic differences of black spruce from white spruce and Sitka spruce [14,19-21], we expect some unique genes in the black spruce transcriptome. Black spruce is an early successional shade-intolerant species whereas white spruce and Sitka spruce are late successional shade-tolerant species. Black spruce can grow in poor conditions, such as bogs, whereas white spruce grows on well-drained soils. These species-specific traits affirm a need to sequence and characterize the black spruce transcriptome.

Much of the EST sequencing in conifers has been performed using wood forming tissues and secondary xylem due to the economic importance of wood [1,2,4,22-25]. There are a number of studies that have sequenced transcripts from needle tissues, including: lodgepole pine (*Pinus contorta*) [6], sugar pine (*Pinus lambertiana*) [24], loblolly pine [24], maritime pine (*Pinus pinaster*) [23] and Norway spruce [26]. These studies have provided some basis for the genes expressed in needle tissue; however, more comparative work is needed to understand their role in important metabolic pathways, such as photosynthesis.

The objective of our study was to develop a black spruce transcript resource, and thus, facilitate structural and functional spruce genomics projects related to growth and adaptation. Here, we report the results of the first EST sequencing project from black spruce in which cDNA clones were isolated and sequenced from a standard cDNA library constructed from needle tissues in 2002. We conducted bidirectional Sanger sequencing of ESTs to produce high quality, long reads to assist with the identification of full-length genes. We assembled ESTs into contigs and singletons, and subsequently performed comparative protein annotations with the non-redundant (NR) protein database and UniGene clusters available at NCBI for model plant species. We further conducted nucleotide similarity analysis with EST sequences available from all major plant species (dbEST), as well as species-specific sequences from various gymnosperms and angiosperms. Gene Ontology terms were assigned and the ESTs were manually evaluated for specific categories, including transcription factors and photosynthetic genes. Finally we used black spruce EST data for the detection of simple sequence repeats (SSRs).

## Methods

### Plant material and cDNA library construction

Total RNA was extracted from 2 g of freshly growing needles of three different black spruce seedlings established in the greenhouse at Dalhousie University, following the protocol described in [27]. Quality and quantity of the isolated RNA were determined using a spectrophotometer (SPECTRAmax PLUS, Molecular Devices Corporation, Sunnyvale, CA, USA) and extracted RNA was found to be of high quality (OD_260_/OD_280_ = 1.82). The quantity of isolated RNA was approximately 120 μg per g of the needle tissue used. The polyA RNA was purified using RNeasy Mini Kit (Qiagen Inc., Mississauga, ON, Canada) following the manufacturer’s instructions. The cDNA library was constructed using Creator smart cDNA library construction kit (Clontech Laboratories Inc. CA, USA). The oligo dT primed cDNA inserts were directionally cloned in pDNR-LIB vector and transformed using XL-10 gold ultra-competent cells of *Escherichia coli*. Plasmid DNA was isolated from the transformed white colonies selected from the overnight grown cells on Luria Broth agar plates containing chloramphenicol (30 μg/ml) using QIAprep Spin Miniprep kit (Qiagen Inc. Mississauga, ON, Canada). The quality and quantity of the isolated plasmid DNA was confirmed on 0.8% agarose gels with known amount of lambda DNA before sequencing.

### cDNA sequencing

Sequencing reactions were performed in a PTC-200 thermal cycler (MJ Research, Reno, NV, USA) using the Thermosequenase fluorescent labeling primer cycle sequencing kit with 7-deaza dGTP (Amersham Pharmacia Biotech, Freiburg, Germany) according to the manufacturer’s instructions. The sequencing products were resolved on a LI-COR 4200 L sequencing system (LI-COR Biosciences, Lincoln, NE, USA). A total of 2,486 cDNA clones were sequenced in both directions using IRD labeled M13F (5΄-AAA CAG CTA TGA CCA TGT TCA-3΄) and M13R (5΄-GTA AAA CGA CGG CCA GT-3΄) primers.

### Preliminary sequence processing

Processing of raw trace files was performed through the customized TreeGenes EST pipeline [28]. Base-calling and quality-assignment of the sequences were conducted with Phred (versions 0.000925.c and 0.020425.c) [29,30]. Low quality bases below Phred20 were masked and vector sequences were trimmed from the ends. The cross_match program was used for this purpose with minmatch 12 and minscore 20 [31]. Sequences with less than 100 high-quality bases (Phred20 or better) after trimming and sequences with polyA tails of ≥ 100 bases were removed from the analysis. The resulting sequence set was compared against the non-redundant (NR) protein database [32] and top ranked BLAST matches to species other than plants with score values > 70 were flagged as contaminants; no such sequences were found in our sequence dataset. The processed sequences were assembled into contigs and singletons using USEARCH v6.0 [33] with 95% identity. EST and contig redundancy was calculated as described in Kirst *et al.*[2]. Simple sequence repeats (SSRs) present in the EST sequences were identified and analyzed using the simple sequence repeat identification Tool (SSRIT) [34]. The parameters were set for detection of perfect di-, tri-, tetra-, and pentanucleotide motifs with a minimum of 10, 7, 5, and 4 repeats, respectively.

### Comparative sequence analysis

The following databases were used to perform BLASTX[32] and BLASTN [32] analyses for annotation of the EST singletons and contigs: 1) *Arabidopsis thaliana*, UniGene Build #74, 30,633 clusters; 2) *Populus* UniGene Build#11, 15,056 clusters; 3) *Oryza sativa*, UniGene Build #86, 44,118 clusters; 4) *Vitis vinifera*, UniGene Build #13, 22,101 clusters; 5) *Physcomitrella patens*, UniGene Build #4, 17,573 clusters; 6) *Pinus* and *Picea*, UniGene Build #13, 61,706 clusters; 7) NR database of GenBank, NCBI release 192, release date October 15, 2012; 8) EST_OTHERS in NCBI download date October 21, 2012; 9) UniProt Plant Protein databank in NCBI download date October 9, 2012. All BLAST searches were subject to an e-value cut-off of 1e - 05. In reporting BLAST results, the BLAST score was used which incorporates both the similarity metric and the e-value to provide a representation of the hit’s uniqueness and overall similarity to the query sequence. BLASTX searches were targeted against model species while BLASTN searches focused on comparisons against conifer species with public sequence resources. In addition to BLAST annotations, the pipeline-directed Gene Ontology (GO) assignments were conducted from applicable results in the categories of Molecular Function and Biological Process. The hierarchical GO structure was stored locally to resolve consistent levels of annotation. In order to classify sequences into comparable categories, InterPro scan wrappers were applied to generate BRENDA enzyme, SignalP, TMHMM, and PFAM protein domain results. Full-length unique ESTs were identified from BLASTX sequence similarity searches. To be considered full-length, sequence were required to have greater than 80% identity and include the start codon for the translated protein.

## Results and discussion

### EST sequence quality, contigs and unique sequences

A sequence read length of >100 bp from either one or both directions was obtained for 2,486 cDNA clones. After sequence processing and removing vector and low quality sequences, a total of 4,594 high quality reads (2,455 5*'* end and 2,139 3*'* end) were obtained (Table 1). The average read-length of ESTs used for sequence analysis was 532 bp. The EST length was distributed from 100 to 500 bp (45%), 501 to 800 bp (43%) and 801 to 1242 bp (12%). Sequence length of greater than 800 bp was obtained for one-eighth of our ESTs.

**Table 1:** Summary of EST sequencing and assembly results

**EST sequences and contigs**	**Number**
Total EST sequences	4,594
Number of 5′ sequences	2,455
Number of 3′ sequences	2,139
Number of contigs	497
Number of singletons	2,234
Average assembled EST length	532.5
Number of full-length cDNA sequences	216
Number of assembled ESTs with:	
Significant BLASTX annotations	1,717
Significant BLASTX annotations with known function	1,478
No BLASTX annotation information	1,014
Average number of sequences per contig	4.75
Number of contigs containing:	
2 ESTs	237
3 ESTs	91
4 ESTs	51
5 ESTs	28
6 ESTs	17
7 ESTs	20
8 ESTs	7
9-11 ESTs	19
12-14 ESTs	9
15-17 ESTs	6
18-20 ESTs	5
22-25 ESTs	3
>25 ESTs	4

**Figure 1:**
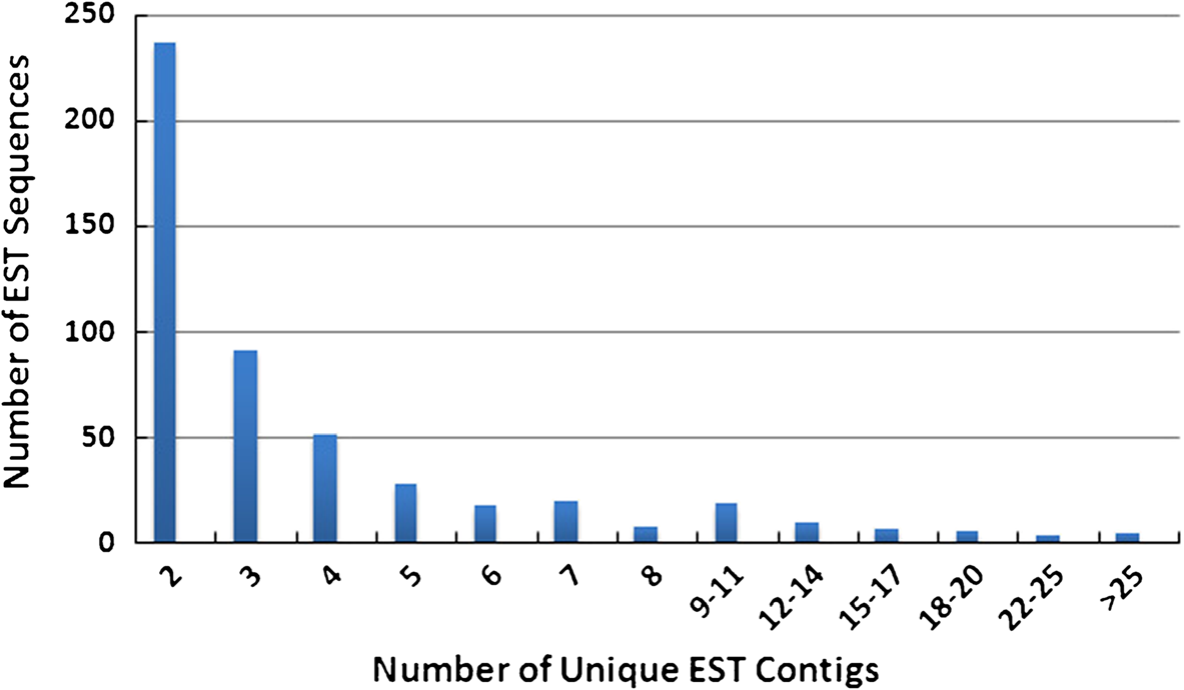
Distribution of individual 5′ and 3′ EST sequences among the clustered contigs.

We identified a total of 2,731 unique sequences, consisting of 2,234 singletons and 497 contigs from the assembly of 4,594 overlapping and contiguous quality reads (Table 1). The average length of singletons, and contigs, was 526 bp, and 560 bp, respectively. The average and maximum number of ESTs in a contig was 4.75, and 208, respectively (Table 1; Figure 1). The average GC content of the unique sequences set is 46.3%. All of the 4,594 high quality EST sequences have been deposited into GenBank under the accession numbers dbEST JZ079173 - JZ083766. They have also been submitted to the TreeGenes database [35].

EST redundancy in the present study was 51%, which is lower than estimates of 59 to 85% reported in other representative conifer studies (Table 2), even though our ESTs were sequenced from a standard non-normalized cDNA library versus normalized cDNA libraries used in most of the other studies. The lower EST redundancy could be due to the fact that relatively small number of sequences from the cDNA library in this study. As the number of ESTs sequenced from a single cDNA library increases, the percent EST redundancy also increases. Inevitable causes of redundancy in cDNA libraries are non-uniform abundance of mRNAs from different genes [36], presence of paralogous genes, and members of multigene families [37]. On the other hand, the redundancy inherent in standard cDNA libraries improves the assemblies as overlapping ESTs from a single gene can be aligned to generate a single contig [36,38,39]. The contig redundancy of 4.7% in our study was similar to that observed in other representative conifer tree species (Table 2). Thus, the quality of our EST sequences appears to be quite high. It should be noted that it is difficult to make comparisons between numbers of transcripts among different projects and genera as they are influenced by the genome and/or transcriptome size of the study species. In addition, these comparisons are influenced by the assembly method [24] and the number of input reads.

**Table 2:** **Comparison of EST sequencing statistics with representative *Picea *** **and *Pinus *** **studies**

**Species**	**Sequencing method**	**Average length, bp**	**Number of ESTs (a)**	**Singletons (b)**	**Contigs (c)**	**No of unigenes (b + c)**	**EST redundancy,% [(a-b)/a]**	**Contig redundancy [(a-b)/c]**	**Reference**
***Picea mariana***	Sanger	532	4,594	2,234	497	2,731	51%	4.7	Current study
***Picea glauca***	Sanger	615	49,101	7,224	9,354	16,578	85%	4.5	[25]
***Picea sitchensis***	Sanger	-	147,146	26,804	19,941	46,745	82%	6.0	[4]
***Pinus taeda***	Sanger	364	59,797	12,307	8,070	20,377	79%	5.9	[2]
***Pinus contorta***	454	306	586,732	239,793	63,657	303,450	59%	5.5	[6]

### Functional gene annotations for unique transcript sequences and gene discovery

Translated nucleotide to protein comparisons were made for the 2,731 *P. mariana* unique sequences (2,234 singletons and 497 contigs) against the non-redundant (NR) protein database. 1,319 (59%) of 2,234 singletons and 398 (80.1%) of 497 contigs, had significant BLASTX hits to known proteins, yielding annotations for 1,717 (62.8%) black spruce unique sequences (Table 1; Additional file 1: Table S1). As expected, the percentage of contigs (80.1%) showing significant similarity with the NR database was higher than singletons (59%). This may be due to greater sequence lengths of the contigs in comparison to shorter singletons. Of the 1,717 annotated unique sequences, 1,478 (54%) represented sequences with known gene functions. In all cases, the most significant, informative annotation was selected. The remaining 239 annotated sequences had annotations that were predicted, hypothetical, or unknown (Additional file 1: Table S1). No contaminants were found after analysis of BLASTX results as the cDNA library was developed from fresh needles of green-house grown seedlings.

A total of 1,014 (37.1%) sequences had no significant BLASTX hits with the NR protein database. The sequence divergence among gymnosperms and angiosperms is a limiting factor for gene annotation in conifers. Similar statistics were obtained for BLASTX similarity analysis of ESTs against publically available databases for white spruce [25] Sitka spruce [4], and Norway spruce [26], which reported no annotations for 15-30% of the transcripts. These results demonstrate that available datasets are not sufficient for annotation of conifer transcripts. In theory, these un-annotated sequences (1,014) could be *P. mariana* specific transcripts or short segments of genes that would be recognized as homologs if more substantial sequences sets were available. Perhaps, these sequences represent regions of proteins that have diverged too much and escaped our similarity search criteria. Finally, these un-annotated sequences could represent partial transcripts with mostly UTRs which, in general, show lower degree of conservation among species. Following the initial analysis of ESTs with the NR database, sequences were annotated against the highly curated plant protein UniProt databank and produced a total of 1,478 significant annotations with known functions (Table 1 and Additional file 1: Table S1). The gene annotations from ESTs in this study represent only a portion of gene repertoire in *P. mariana*, more transcriptome sequencing is needed to identify the needle tissue transcriptome.

Predicted proteins from the first whole genome sequence of Norway spruce have become available [9]. However, the Norway spruce genome assembly and protein predictions are at the very first stage, whereas we have used highly curated and reliable plant protein and NR protein databases for annotation of our black spruce unique contigs and singletons. Thus, the functional annotations reported here, although conservative, should be quite reliable. Also, the first draft genome of white spruce is published [10] but there is no information available on its predicted proteins. In future, with the availability of advance generations of Norway spruce genome assembly and identification of functional proteins, the black spruce unique transcriptome sequences should be analyzed against the Norway spruce and other conifers (if available) functionally analyzed and predicted proteins. This may provide information if an EST is a member of a longer protein that is actually or predictably expressed.

**Figure 2:**
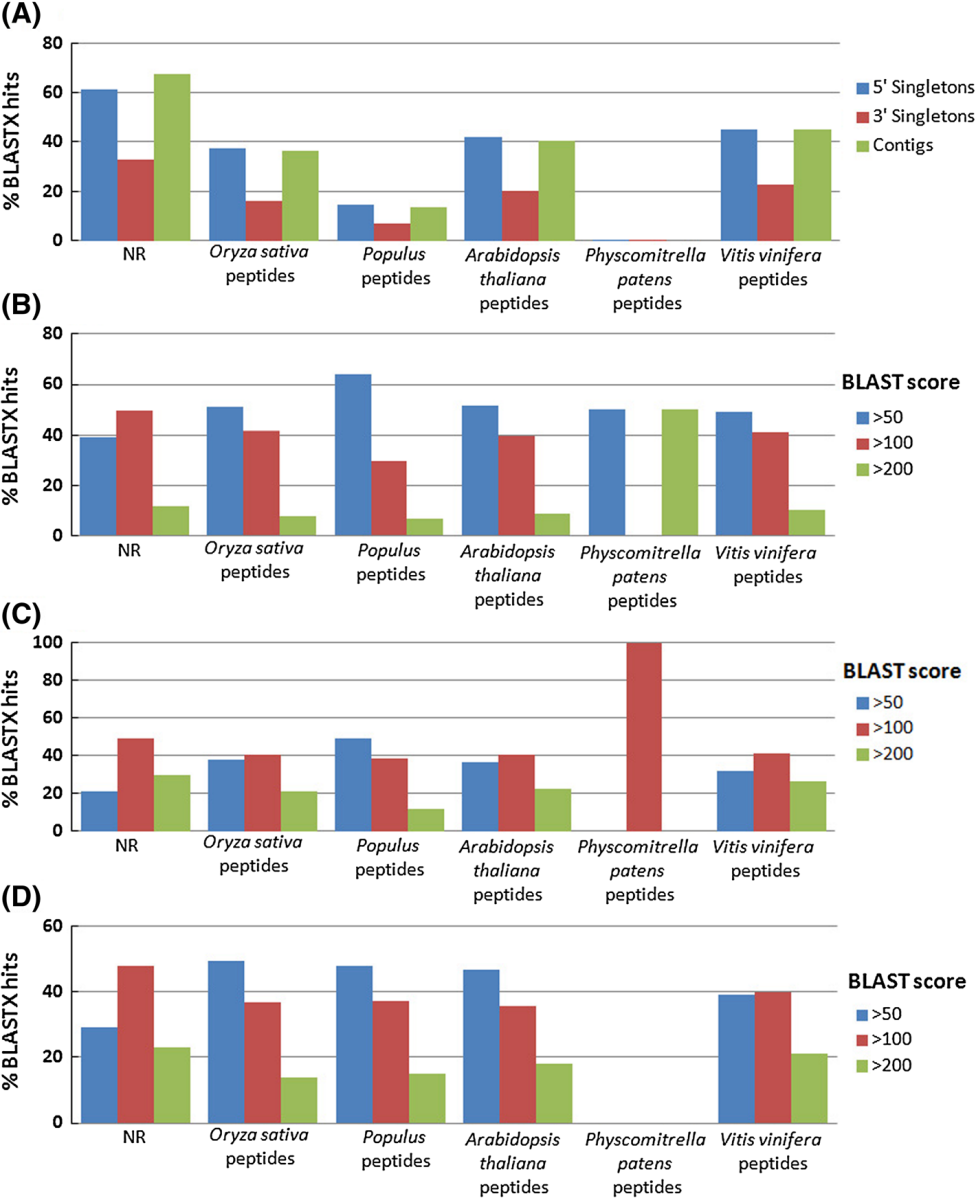
**Conservation between *Picea mariana *unique sequences and peptides from five model plant species and non-redundant protein database: BLASTX searches were performed against: *Arabidopsis thaliana, Populus, Vitis vinifera, Oryza sativa*, and *Physcomitrella patens*. (A)** Percentage of *Picea mariana* unique sequences showing similarity with peptides from six databases (> 50 score). Contigs showed the highest number of hits to all five model plant species databases followed by 5*'* singletons and then 3*'* singletons. **(B**-**D)**. 3*'* singletons, 5*'* singletons and contigs were analyzed separately using low (score > 50), medium (score > 100) and high (score > 200) BLAST stringency thresholds. **(B)** The 3*'* EST singletons had a much greater number of annotations with the model databases in the > 50 score category ranging from 49% to 64%. **(C)** The 5*'* EST singletons had the greatest number of hits with scores > 100. **(D)** The EST contigs had the largest number of high scoring hits (> 50) with representation across most of the five model plant species databases.

The BLASTX comparisons of *P. mariana* 3*'* singletons, 5*'* singletons, and contigs were also conducted against the protein sequences of five sequenced plant genomes (*Arabidopsis*, *Populus*, *Oryza sativa*, and *Vitis vinifera*) and moss (*Physcomitrella patens*) (Figure 2; Additional file 2: Table S2). In all species, contigs and 5*'* singletons showed higher similarity (0-45%) with the species-specific peptides than 3*'* singletons (0.2-23%) (Figure 2A). The 3*'* singletons obtained the most annotations with a BLAST score range from 50–99. Between 49% and 64% of the 3*'* singletons contained an annotation with *Arabidopsis, Populus*, *Oryza sativa*, *Vitis vinifera*, and *Physcomitrella patens* at score range of 50–99 (Figure 2B). However, 5*'* singletons had a larger percentage of BLAST hits with a score range of 100–199 (Figure 2C). Contigs and 5*'* singletons, had the best alignments with scores >200 in all model plant species queried (Figure 2C,D). The greatest number of black spruce contig annotations, with a score range of 50–99 (~50%), were obtained from the comparisons with the *Populus* and *Oryza sativa* proteins. A previous study involving 5*'* singletons, 3*'* singletons, and contigs for white spruce and Sitka spruce, showed a higher percentage of BLASTX similarity with the same angiosperm species as in our study [4]. The percentage of white spruce and Sitka spruce 5*'* singletons (74-82%) showing BLASTX similarity was higher than 3*'* singletons (60-68%) [4]. In contrast, only 49.7% of loblolly pine ESTs showed significant BLASTX hits with *Arabidopsis* peptides [2]. Based on the functional gene annotation analysis, *P. mariana* transcripts showed higher annotation rates with angiosperms peptides used in the present study than with moss peptides. It may be due to the fact that conifers are phylogenetically closer to angiosperms than to mosses [40]. Black spruce transcripts are expected to show the highest annotations with highly curated spruce and pine peptide database when it becomes publicly available.

### Gene ontology classification, full-length genes, and gene families

**Figure 3:**
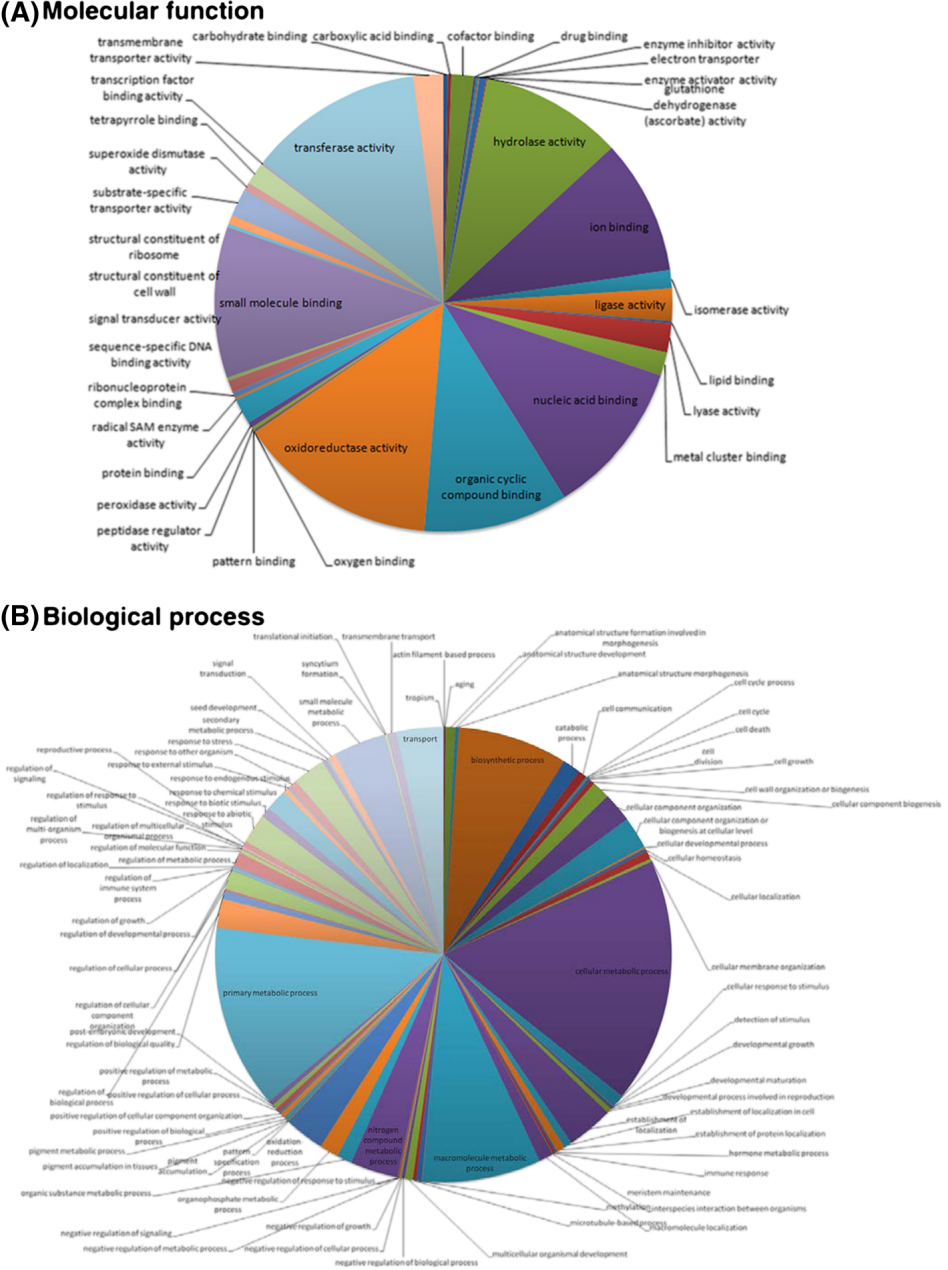
**Gene ontology classification of *Picea mariana *unique sequences: BLASTX at an e-value cut-off of 1e - 05 were performed against UniProt’s plant protein databank.** This was used in conjunction with InterProScan to identify domains linked to GO terms. Terms were standardized to hierarchy level 4 and 5 in order to compare the results across the annotations. **(A)** A total of 533 unique sequences were associated with at least one molecular function term. **(B)** A total of 572 unique sequences were associated with at least one biological process term.

Gene Ontology (GO) results were assigned to describe the functional distribution within the EST unique sequences derived from *P. mariana* needle tissue (Figures 3A and B; Additional file 1: Table S1). A total of 533 unique sequences were associated with at least one molecular function term and 572 sequences were associated with at least one biological process term (Figures 3A and B; Additional file 1: Table S1). Molecular function assignment revealed that oxidoreductase activity, transferase activity, ion binding and nucleic acid/nucleotide binding accounted for the largest portion of the *P. mariana* unique genes identified (~47% combined). Among the ion binding proteins, the majority were small metal ion binding proteins such as metallotheonins, zinc and calcium binding proteins. These genes are involved in stress response [41] and protect cells from toxic metal and assist in metal transport [42,43]. Metallothioneins are able to sequester excess amounts of metal ions, and participate in homeostasis and antioxidant functions [44]. Various nucleic acid binding proteins were also targeted with high frequency as they are important components of transcriptional machinery of cells [45]. Previous study involving *Quercus* spp. contigs also reported nucleotide binding as abundant GO term in terms of molecular function [13]. Other significant categories included hydrolase activity, small molecule binding and organic cyclic compound binding (~10% each) (Figure 3A). It is worth noting that although the EST number in our study is relatively small, genes involved in 36 molecular functions could be identified.

Biological process describes the major biochemical pathways that the sequences may be involved in (a much higher resolution than molecular function). The primary categories in our study include cellular metabolic process (17%), primary metabolic process (13%), macromolecule metabolic process (8%), and biosynthetic process (8%) (Figure 3B). The results from the functional distribution highlight that transcripts from diverse categories are represented in *P. mariana* unique sequences. The molecular functions and biological processes assigned for the black spruce unique contigs and singletons are consistent with the metabolic pathways active during vigorously-growing black spruce seedlings in the greenhouse conditions from which samples for RNA preparation were collected. The molecular function and biological process Gene Ontology terms are also consistent with the similar results reported in other studies that used needles for EST or transcriptome sequencing [6,23,24,26].

A combination of BLAST annotations, GO terms, and supplemental InterProScan data was used to generate lists of gene families of interest. Two of the groups highlighted here include transcription factors (Table 3) and genes involved in the photosynthetic pathway (Additional file 3: Table S3). Transcription factors are DNA-binding sequence-specific proteins that interact with the promoter sequences of target genes and modulate the gene expression [46]. A total of 24 putative transcription factor sequences were identified based on the GO term assignment of *P. mariana* unique sequences. Majority of the transcription factors were represented by myc, myb, and WRKY domains. These transcription factors families have also been reported in the white spruce EST collection [18,25]. They are key regulators of various biological processes involved in growth and development [47,48]. Also, they are expressed in response to various biotic and environmental stresses encountered by needles during their life cycle [48,49]. Some WRKY proteins have been found to be involved in the signal transduction pathway [49].

**Table 3:** EST singletons and contigs annotated as putative transcription factors

**EST contig/singlet**	**Annotation sequence identifier**	**Annotation description**
estPama_needle_Contig7	gi|356520192|ref|XP_003528748.1|	Transcription factor 25-like
estPama_needle_Contig151	gi|225438387|ref|XP_002275126.1|	RNA polymerase II transcriptional coactivator KIWI
estPama_needle_2137-700_5	gi|297794475|ref|XP_002865122.1|	Myb family transcription factor
estPama_needle_2089-700_5	gi|357125278|ref|XP_003564322.1|	Transcription elongation factor 1 homolog isoform
estPama_needle_BSc1-839-700_5	gi|356500773|ref|XP_003519205.1|	Transcription initiation factor IIA subunit 2-like
estPama_needle_Contig232	gi|357144617|ref|XP_003573355.1|	Transcription factor ILR3-like
estPama_needle_Contig326	gi|255547594|ref|XP_002514854.1|	Transcription factor, putative
estPama_needle_Contig314	gi|358346858|ref|XP_003637481.1|	Transcription factor
estPama_needle_Contig320	gi|115470937|ref|NP_001059067.1|	Similar to C-Myc binding protein
estPama_needle_BSC1-987-800_3	gi|225461347|ref|XP_002281902.1|	PAP-specific phosphatase HAL2-like
estPama_needle_1584-700_5	gi|115470937|ref|NP_001059067.1|	Similar to C-Myc binding protein
estPama_needle_1817-700_5	gi|225437251|ref|XP_002282315.1|	Thylene-responsive transcription factor 7-like
estPama_needle_2311-700_5	gi|302840754|ref|XP_002951923.1|	Transcription factor jumonji domain-containing protein
estPama_needle_1788-700_5	gi|15233968|ref|NP_195574.1|	Transcription repressor MYB4
estPama_needle_Contig490	gi|255547594|ref|XP_002514854.1|	Transcription factor, putative
estPama_needle_BSc1-785-700_5	gi|255585312|ref|XP_002533354.1|	WRKY transcription factor, putative
estPama_needle_BSC1-935-800_3	gi|225438387|ref|XP_002275126.1|	RNA polymerase II transcriptional coactivator KIWI
estPama_needle_1371-700_5	gi|255561893|ref|XP_002521955.1|	Associate of C-myc, putative
estPama_needle_2143-800_3	gi|357494843|ref|XP_003617710.1|	mTERF domain-containing protein
estPama_needle_2512-700_5	gi|359494595|ref|XP_002262881.2|	Nuclear transcription factor Y subunit C-9
estPama_needle_1540-700_5	gi|334183649|ref|NP_001185317.1|	Transcription elongation factor SPT6
estPama_needle_2467-700_5	gi|302840754|ref|XP_002951923.1|	Transcription factor jumonji domain-containing protein
estPama_needle_BSc1-839-800_3	gi|255572854|ref|XP_002527359.1|	Transcription initiation factor iia (tfiia), gamma chain, putative
estPama_needle_BSc1-781-700_5	gi|356547095|ref|XP_003541953.1|	WRKY transcription factor 6

Since the cDNA library was constructed from needle tissues, genes related to photosynthesis were expected to be abundantly expressed. A total of 232 *P. mariana* unique transcripts related to photosynthetic pathway were identified (Additional file 3: Table S3). This group included chlorophyll a/b binding protein, light-harvesting complex proteins, photosystem I and II reaction center proteins, ribulose bisphosphate carboxylase (RuBisCo), oxygen-evolving enhancer protein, granule-bound starch synthase and nucleoside diphosphate. In plants, RuBisCo is more abundant during the day when it is transcriptionally regulated by the light receptor phytochrome, thus fixing carbon dioxide in photosynthesis [50]. We collected needle samples for RNA extraction during daytime.

**Figure 4:**
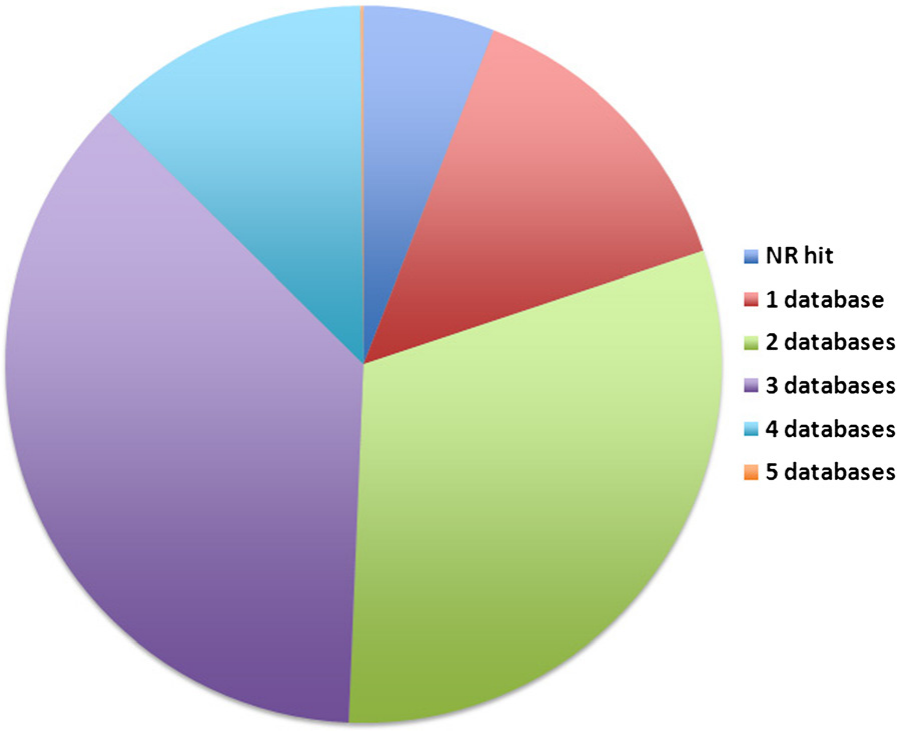
Distribution of full-length cDNAs according to BLASTX hits in the number of species-specific repositories: Of the 216 full-length genes identified, the majority (90%) had significant annotations in all five datasets at an e value (expected value) cut-off of 1e - 05.

BLAST results against individual species as well as the NR repository provided details on the 216 full-length ESTs identified (Additional file 4: Table S4). Of these, 32% had annotations in three of the five model plant species (Figure 4). The 216 full-length gene sequences were represented by 67 contigs, 131 5*'* singletons, and 18 3*'* singletons. The full-length cDNA sequences are an important resource for characterization of individual genes and members of multigene families, as well as for functional and comparative genomics. Additionally, they are essential resources for downstream annotation of related conifer genomic sequences.

### Gene expression and highly abundant transcripts

A preliminary estimation of gene expression can be given by the frequency distribution of the ESTs representing a gene in the library. Among the annotated sequences, 30 contained at least 10 supporting ESTs (Table 4). A family of lipid-transfer protein (represented by 77 ESTs) was most abundant. They are known to be involved in biotic and abiotic stress responses [50]. Additionally, transcripts related to Bet v I allergen family, antimicrobial protein, germin-like protein, and many photosynthetic pathway genes were also highly abundant. The supporting ESTs represent between 1 and 9 unique contigs (Table 4; Additional file 5: Table S5). The supplemental Additional file 5: Table S5 provides details on 149 annotated sequences with supporting ESTs. The examination of the complete set shows that several growth factors, disease responsive genes, transcription factors, and photosynthetic mechanism proteins are represented by multiple ESTs (Table 4; Additional file 5: Table S5). These may be the result of multigene families, in addition to the inherent redundancy of ESTs in non-normalized cDNA libraries [37].

**Table 4:** Estimation of gene expression: unique EST sequences with > 10 ESTs

**Putative protein identification**	**Number of unique EST sequences**	**Number of ESTs**
Non-specific lipid-transfer protein	10	77
Bet v I allergen family protein. (Os04t0465600-01)	2	40
Ribulose bisphosphate carboxylase small chain 1A	4	35
Antimicrobial peptide 1	2	30
Non-protein coding transcript. (Os07t0139600-01), partial	9	28
Histone H3	4	24
Light-harvesting complex	4	24
Germin-like protein 8-14-like	2	21
Metallothionein-like protein 3B-like	6	21
Photosystem I reaction center subunit V	2	21
Photosystem II subunit X	3	21
Cell wall-associated hydrolase, partial	8	20
LOW QUALITY PROTEIN: photosystem II 10 kDa Polypeptide, chloroplastic-like	1	18
Translation machinery associated protein TMA7	2	17
Photosystem I reaction center subunit N, chloroplast precursor, putative	2	15
Protein ralf-like 34	3	15
Transmembrane protein TPARL, putative	1	13
Hypothetical protein BrnapMp036 (mitochondrion)	3	13
Hypothetical protein EAAG1_11607	1	13
ATP synthase subunit beta	3	11
Metallothionein-like protein 2-like isoform 2	1	11
Ribosomal protein S14 (chloroplast)	3	11
Auxin-binding protein ABP19a precursor, putative	1	10
Photosystem II 5 kDa protein, chloroplastic-like	1	10
Similar to Anth (Pollen-specific desiccation-associated LLA23 protein). (Os11t0167800-01)	2	10
Chlorophyll a-b binding protein M9, chloroplastic precursor	1	10
Photosystem I subunit O	2	10

High abundance of genes involved in photosynthesis, growth and transcription factors is quite expected because the cDNA library was constructed from needles of actively growing black spruce seedlings in the greenhouse conditions. Abundance of stress and disease responsive genes expressed in black spruce seedlings growing under optimal greenhouse conditions suggest that these genes are also involved in plant functions other than their response to abiotic and biotic stress. Nevertheless, the stress and disease responsive genes identified in our study provide very valuable transcriptomic resource for structural and functional genomics studies in black spruce.

### Sequence similarities, life history and ecological traits and evolutionary relationships

**Figure 5:**
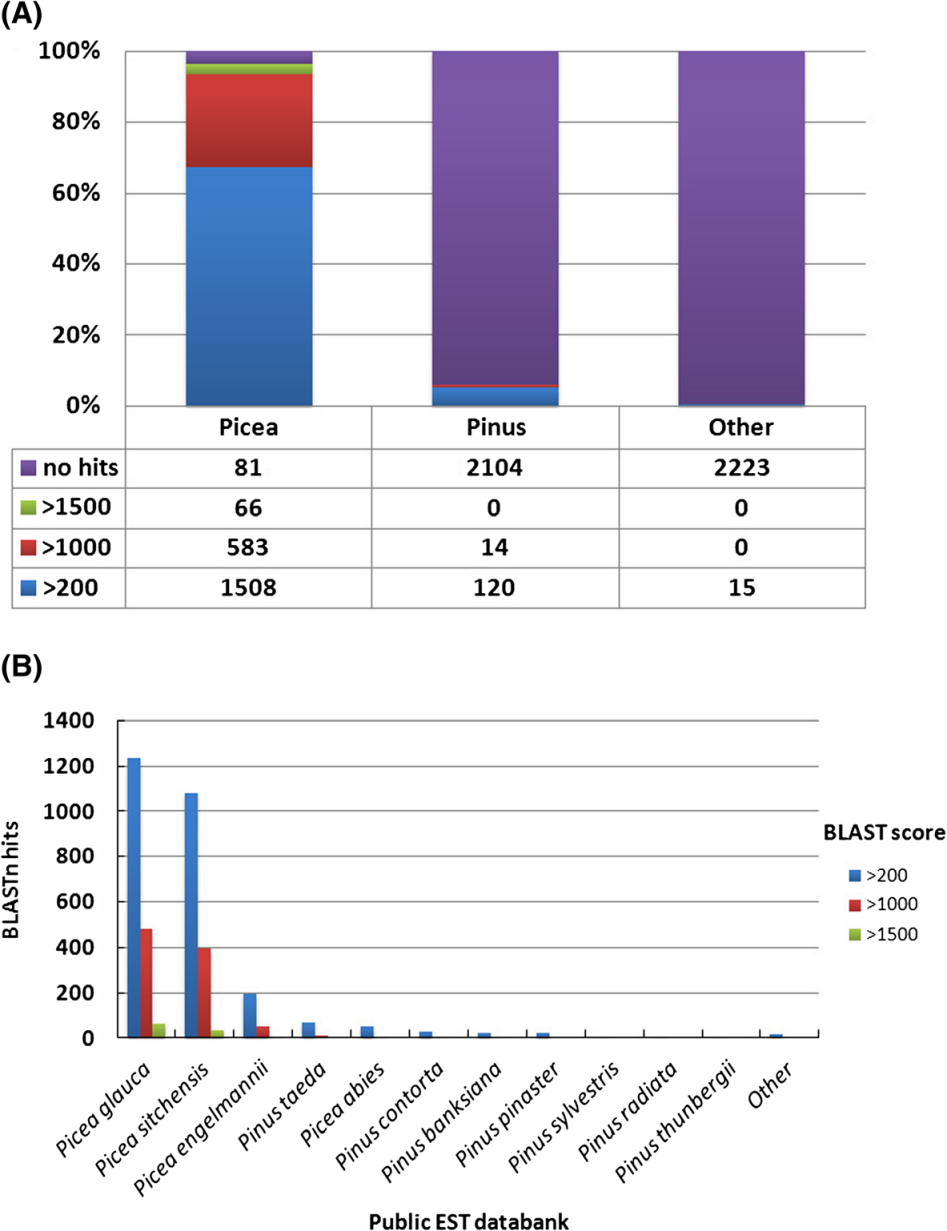
**Conservation between *Picea mariana *unique sequences and cDNA sequences from other conifer species: (A) BLASTN searches were performed against NCBI’s est_others repository and those with hits with a minimum e-value of 1e - 05 were organized into *Picea*, *Pinus *and Other to examine similarity by genus.** 18% of the sequences (493) had no hits within *Picea* and are therefore likely to be unique to *Picea mariana*. **(B)** The same BLASTN searches were divided by species and organized into categories by score: > 200, > 1,000, and > 1,500. The greatest number of hits was observed with *Picea glauca* and *Picea sitchensis.* These two species also had the greatest number of nearly identical hits (scores > 1500).

Unique sequences were also compared with ESTs from major plant species combined (dbEST database of GenBank, excluding ESTs reported in this study) using BLASTN. As expected, sequence similarity between the *P. mariana* sequences and published gymnosperm ESTs was high (Figure 5; Additional file 6: Table S6). Results were examined first by genus (Figure 5A) and subsequently by specific species within *Picea* and *Pinus* (Figure 5B). A total of 493 sequences did not have a significant BLASTN hit and are therefore considered to be novel sequences. These novel sequences may represent transcripts specific to *P. mariana* related to its species-specific traits. As noted earlier, *P. mariana* differs from *P. glauca*, *P. sitchensis* and *P. engelmannii* for many life history, growth, morphological, ecophysiological, adaptive and insect resistance traits [14,19,20]. Black spruce is an early successional pioneering species, whereas white and Sitka spruces are late-successional and climax species. It is a slower growing species than white and Sitka spruce. Black spruce unlike white and Sitka spruce can grow on poorly drained, wet organic, loamy clay nutrient-poor soils, with low soil temperatures and high moisture contents [14,19,20]. Black spruce is less susceptible to some insect diseases, such as spruce budworm (*Choristoneura fumiferana*), than white spruce [14]. White spruce is very closely related to Engelmann spruce and Sitka spruce, and hybridizes naturally with these species in the zone of overlap [19]. Phylogenetically, black spruce is distinct from white, Sitka and Engelmann spruces [21]. Occurrence of some species-specific genes in *P. mariana* is therefore expected. It is noteworthy that despite the relatively smaller *P. mariana* EST dataset as compared to *P. glauca*, and *P. sitchensis*, a significant number of novel transcripts could be detected in our study. This number may be an over-estimate as some of these novel transcripts may be gene segments or regions from 5*'* or 3*'* ends of genes sufficiently diverged to escape our similarity criteria.

Among the remaining, 2,238 sequences, 96% had a hit to a member of the *Picea* genus and 6% had significant similarities to a member of the *Pinus* genus (Figure 5A). When viewing the results by species, *Picea glauca* and *Picea sitchensis* had the majority of significant matches, with more than 65% of the sequences generating a BLAST score > 200 (Figure 5B). These similarity results suggest that the majority of *P. mariana* genes discovered are homologues (orthologs) of other *Picea* species genes and may have originated from a common ancestor. The significant similarities with *P. glauca* ESTs is not surprising as both species are sympatric transcontinental boreal species which can hybridize naturally, although rarely [51]. Within *Pinus*, the greatest number of hits was observed with *Pinus taeda* (68 unique sequences) as expected since the EST resource generated for that species is very large (328,662). *Pinus contorta* followed with 30 unique sequence similarities with scores > 200 (Figure 5B). Only 17 (0.6%) sequences had significant similarity to another plant species outside of the *Picea* and *Pinus* families. These 0.6% BLAST hits to distant species may represent sequences not well characterized in closely related conifer species.

### Simple sequence repeats

A total of 57 different di-, tri-, tetra-, and penta-nucleotide repeats were identified among the *P. mariana* ESTs (Table 5). These were represented by 12 dinucleotide, 39 trinucleotide, three tetranucleotide, and three pentanucleotide repeats. Dinucleotide motifs were the most frequent (72%), followed by trinucleotide motifs, which constitute 26% of total number of SSRs. Among the dinucleotide and trinucleotide repeats AT, and CAG motifs, respectively, were the most abundant. SSR markers have been developed from some of these SSR-containing sequences and mapped on a black spruce genetic map [52]. The SSR markers developed and other that can be developed in the future from the black spruce EST sequences reported here provide a highly valuable resource for various population and conservation genetic studies in black spruce and other conifers.

**Table 5:** Types and distribution of simple sequence repeats

**Type of repeat**	**Repeat motif**	**% of sequences having repeat motif**
Pentanucleotide repeats	(CGCAG)4	0.07
	(TCAGA)4	0.07
	(TGGTC)4	0.07
Tetranucleotide repeats	(ACAT)4	0.07
	(CCTG)5	0.07
	(TAAT)4	0.07
Trinucleotide repeats	(AAC)4	0.15
	(AAG)4	0.15
	(AAT)4	0.07
	(ACG)4	0.07
	(AGA)4	0.07
	(AGC)4or6	0.11 or 0.07
	(AGG)4or5	0.22 or 0.07
	(ATA)5	0.07
	(ATC)4	0.11
	(ATG)4	0.11
	(CAC)5or6	0.07 or 0.07
	(CAG)4or5or6	0.15 or 0.11 or 0.07
	(CAT)4	0.11
	(CCA)5	0.07
	(CCG)4	0.07
	(CGC)5	0.07
	(CGG)4	0.07
	(CTC)4or5	0.07 or 0.11
	(CTG)4or5	0.11 or 0.11
	(CTT)4or6	0.11 or 0.07
	(GAA)4or5	0.22 or 0.07
	(GAC)8	0.07
	(GAG)4or8	0.11 or 0.07
	(GAT)4	0.07
	(GCA)4	0.15
	(GCC)6	0.07
	(GCT)4	0.11
	(GGA)4or5	0.22 or 0.07
	(TAA)4	0.07
	(TAG)4	0.07
	(TAT)5or6	0.07 or 0.07
	(TCA)4	0.11
	(TCC)4or5	0.15 or 0.07
	(TCT)4or5	0.11 or 0.15
	(TGA)4	0.07
	(TGC)4	0.07
	(TGG)6	0.07
	(TTC)4	0.26
	(TTG)4	0.11
Dinucleotide repeats	(AC)4or5or6	0.77 or 0.18 or 0.11
	(AG)4or5	1.39 or 0.15
	(AT)4or5or6or7or8	2.27 or 0.70 or 0.11 or 0.15 or 0.15
	(CA)4or5or6	0.81 or 0.07 or 0.11
	(CG)4or5or6	0.29 or 0.11 or 0.07
	(CT)4or5	0.84 or 0.18
	(GA)4or5or6	0.99 or 0.07 or 0.18
	(GC)4or5or6	0.40 or 0.15 or 0.07
	(GT)4or5or6	0.37 or 0.07 or 0.07
	(TA)4or5or6or7	2.05 or 0.37 or 0.11 or 0.11
	(TC)4or5or6	1.28 or 0.22 or 0.07
	(TG)4or5	0.73 or 0.15

## Conclusions

We report here the first EST resource of high quality for a widely-distributed, ecologically and economically important boreal conifer, black spruce. Despite the relatively small number of EST sequences compared to *Picea glauca* and *P. sitchensis*, our study identified 493 novel transcripts with no nucleotide similarity with dbEST, and therefore, represent important addition to dbEST. We have identified genes involved in 36 molecular functions and 90 biological processes. Genes involved in stress response, photosynthetic pathway and growth were most abundant in the ESTs. We have identified 216 full-length genes, ranging from 18 to 265 amino acids in length. The sequences showed the greatest similarities to ESTs from the congeneric and sympatric species, *Picea glauca*. Black spruce ESTs containing 57 different di-, tri-, tetra-, and penta-nucleotide repeats were identified. These sequences could be used for the development of microsatellite DNA markers.

The ESTs, and their annotations provide a valuable genomics resource to the forest tree genomics community in specific and plant genomics community in general. Markers developed from some of the EST sequences have already been mapped on a black spruce genetic linkage map [52]. The ESTs reported will provide an excellent resource for future assembly and annotation of transcriptome sequences from the NGS platforms, as well as for annotation of the spruce whole genome sequences. A comparison of 454 and Sanger reads showed that Sanger reads can improve the assembly and annotation of the 454 datasets [13,53].

### Availability of supporting data

All of the 4,594 high quality EST sequences have been deposited into GenBank under the accession numbers dbEST JZ079173 - JZ083766. They have also been submitted to the TreeGenes database admits multiple queries on the occurrence of ESTs in the library and their functional annotation.

## Abbreviations

ESTs: Expressed sequence tags; NR: Non-redundant; GO: Gene ontology; dbEST: EST database; SSRs: Simple sequence repeats; NGS: Next-generation sequencing.

## Competing interests

The authors declare that they have no competing interests.

## Authors’ contributions

IKM was partly involved in library construction, Sanger sequencing, quality assurance, sequence analysis and processing, and prepared the first draft of the manuscript. JW carried out sequence assembly, annotation, submitted sequences to public databases, and contributed to manuscript preparation; OPR is the Principal Investigator of the Spruce Genomics Project and contributed to the project conception, secured project funding, provided overall project direction and guidance, and contributed to the manuscript preparation and revision. All authors read and approved the final manuscript.

## Supplementary Material

Additional file 1: Table S1 Detailed annotation of complete set of black spruce singletons and contigs. Click here for file

Additional file 2: Table S2 BLASTX similarity results against peptides from five model species: *Arabidopsis*, *Populus*, *Oryza sativa*, and *Vitis vinifera*, and *Physcomitrella patens*. Click here for file

Additional file 3: Table S3 EST singletons and contigs annotated as putative photosynthetic pathway genes. Click here for file

Additional file 4: Table S4 Predicted annotation, GenBank accession numbers for the black spruce predicted full length genes. Click here for file

Additional file 5: Table S5 Estimation of gene expression: Unique EST sequences with 1–9 ESTs. Click here for file

Additional file 6: Table S6 Sequence similarity results for back spruce unique sequences against dbEST. Click here for file
